# Improve service coordination and delivery in community hubs serving homeless and at-risk populations: Hala Shamaa

**DOI:** 10.1093/eurpub/ckac130.002

**Published:** 2022-10-25

**Authors:** H Shamaa, W Sun, V Peters, L Martignetti, E Valant, D Sparks

**Affiliations:** 1 Faculty of Health Sciences, Ontario Tech University, Oshawa, Canada; 2 Social Services, Regional Municipality of Durham, Oshawa, Canada; 3 Office of the Regional Chair and CAO, Regional Municipality of Durham, Whitby, Canada

## Abstract

**Background:**

Two community hubs are currently located in Durham Region, Ontario, Canada, to provide a single point of access to a wide range of support services for individuals experiencing homelessness and other at-risk populations. The community hub in Oshawa is formally known as the Back Door Mission for the Relief of Poverty and the community hub in Ajax is formally known as the Ajax Hygiene Hub. It is unclear if these two community hubs are effective in addressing the needs of individuals experiencing homelessness and how the COVID-19 pandemic continues to impact these services amongst this population. This study was conducted to identify gaps and barriers within the community hub models as well as provide recommendations to improve the coordination and delivery of services serving individuals experiencing homelessness and other at-risk populations.

**Methods:**

A mixed methods approach was utilized in this study, which included surveys for individuals experiencing homelessness, through open-ended and close-ended questions to assess their experiences at either one of the two community hubs. A total of 75 surveys were completed by the study participants (40 surveys in Oshawa and 35 in Ajax). Thematic analysis was performed for all the open-ended survey responses. A literature review was also conducted to evaluate the community hub models as well as best practices for the implementation locally, nationally, and internationally.

**Results:**

Data analysis for the open-ended survey responses revealed the need for housing support, increased resources for medical services, and the expansion of programs provided by the community hubs.

**Conclusions:**

Homelessness is a major public health issue however community hubs play a pivotal role in addressing this concern in Durham Region. The equitable access to a diverse range of services that are co-located in a community hub is imperative for individuals experiencing homelessness, especially during the COVID-19 pandemic.

**Key messages:**

- The needs of homeless populations, program outcomes, service gaps, partnership collaboration, and best practices were explored to help inform the development of new community hubs in Durham Region.
- Study highlighted the effectiveness of community hubs in addressing the needs of homeless populations, whether it should become a permanent solution, and be developed in Durham Region and beyond.

